# Supplementary material for: Incoherent nonadiabatic to coherent adiabatic transition of electron transfer in colloidal quantum dot molecules
Source: Nat Commun. 2023 May 27;14:3073. doi: 10.1038/s41467-023-38470-0 (PMC10224918; doi:10.1038/s41467-023-38470-0)
Supplement: Supplementary file 1 — Supplementary Information [file 41467_2023_38470_MOESM1_ESM.pdf]

Supplementary Information: Incoherent Nonadiabatic to Coherent Adiabatic Transition of Electron Transfer in Colloidal Quantum Dot Molecules

Bokang Hou[1], Michael Thoss[2], Uri Banin[3], Eran Rabani[1,4,5*]

[1] *Department of Chemistry, University of California, Berkeley, California 94720, United States.*

[2] *Institute of Physics, University of Freiburg, Hermann-Herder-Straße 3, 79104 Freiburg, Germany.*

[3] *Institute of Chemistry and the Center for Nanoscience and Nanotechnology, The Hebrew University of Jerusalem, 91904 Jerusalem, Israel.*

[4] *Materials Sciences Division, Lawrence Berkeley National Laboratory, Berkeley, California 94720, United States.*

[5] *The Raymond and Beverly Sackler Center of Computational Molecular and Materials Science, Tel Aviv University, Tel Aviv 69978, Israel.**

---

* Corresponding author. Email: eran.rabani@berkeley.edu

Supplementary note 1: Nanostructure configurations

The core-shell colloidal quantum dot (CQD) nanocrystals (NCs) were constructed by adding CdS shells to a CdSe core which was cleaved from a large crystal with a lattice constant of bulk wurtzite CdSe ($a = 4.3$ Å, $c = \sqrt{\frac{8}{3}}a$). The CQD dimers were then constructed by attaching two NCs either through the [100] symmetric or [001] asymmetric crystal plane indexed by the cubic lattice system as shown in Supplementary Figure 1. [100] orientation results in a mirror plane perpendicular to the attachment direction, while [001] orientation does not. The neck/bridge of the dimer can then be widened by adding additional CdS layers to the connection area between two NCs. Tables. S1 and S2 below summarize the different combinations of D_{neck}, D_{shell} and D_{core} in two orientations used in this study. The structures were minimized with Stillinger-Weber force field parameterized for II-VI nanostructures [1] using the conjugate gradient minimization implemented in LAMMPS [2].

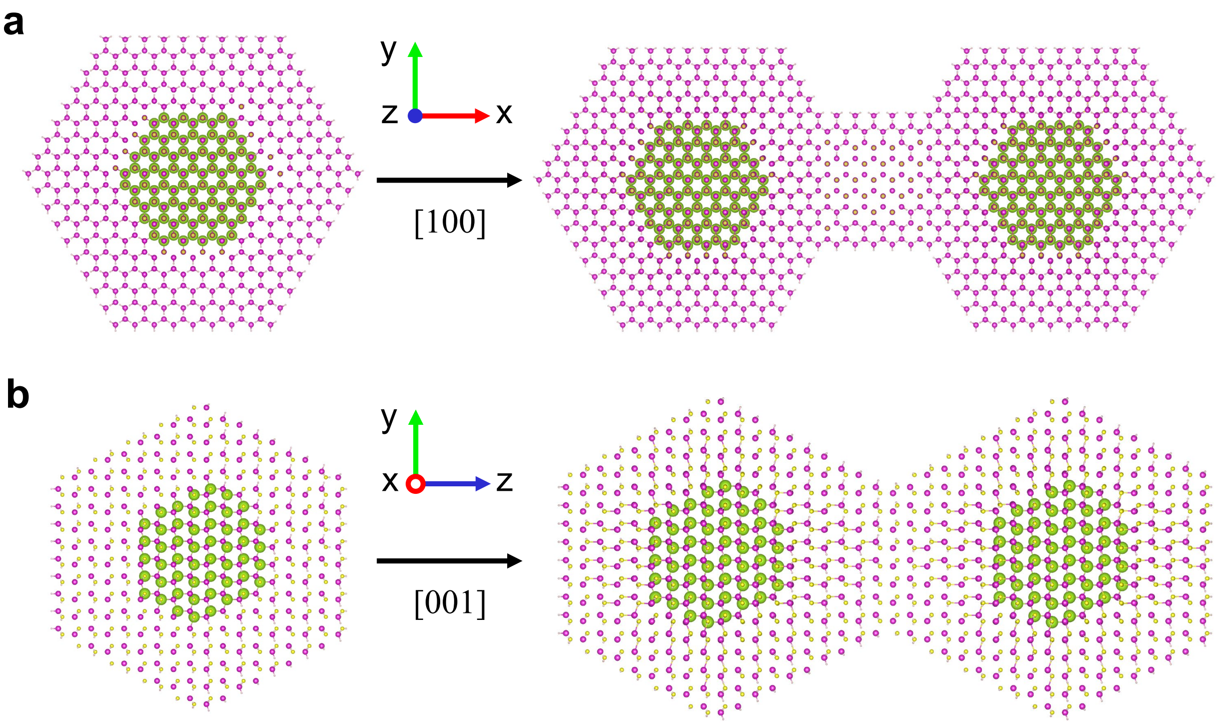

Supplementary Figure 1: Constructing QD dimers through two orientations. **a**, **b** Symmetric QD dimers can be constructed by attaching two CdSe-CdS core-shell quantum dots through [100] orientation (**a**), while asymmetric QD dimers are attached through [001] orientation (**b**). Magenta, yellow, and green colors represent Cd, S, and Se atoms. The size of Se atom is not drawn to scale for illustration purpose.

Orientations	Label	D_{core}	D_{shell}	D	D_{neck}	Formula
[100]	**a**	2.2	1.7	5.7	2.4	$Cd_{5330}Se_{504}S_{4826}$
	**b**	3.0	1.3	5.7	2.4	$Cd_{5330}Se_{966}S_{4364}$
	**c**	3.9	0.9	5.7	2.4	$Cd_{5330}Se_{216}S_{5116}$
	**d**	2.2	1.7	5.7	3.1	$Cd_{5510}Se_{216}S_{5294}$
	**e**	3.0	1.3	5.7	3.1	$Cd_{5510}Se_{504}S_{5006}$
	**f**	3.9	0.9	5.7	3.1	$Cd_{5510}Se_{966}S_{4544}$
	**g**	2.2	1.7	5.7	3.8	$Cd_{5764}Se_{216}S_{5548}$
	**h**	3.0	1.3	5.7	3.8	$Cd_{5764}Se_{504}S_{5260}$
	**i**	3.9	0.9	5.7	3.8	$Cd_{5764}Se_{966}S_{4798}$
[001]	**a**	2.2	1.7	5.7	2.8	$Cd_{5202}Se_{216}S_{4986}$
	**b**	3.0	1.3	5.7	2.8	$Cd_{5202}Se_{504}S_{4698}$
	**c**	3.9	0.9	5.7	2.8	$Cd_{5202}Se_{966}S_{4236}$
	**d**	2.2	1.7	5.7	3.5	$Cd_{5283}Se_{216}S_{5067}$
	**e**	3.0	1.3	5.7	3.5	$Cd_{5283}Se_{504}S_{4779}$
	**f**	3.9	0.9	5.7	3.5	$Cd_{5283}Se_{966}S_{4317}$
	**g**	2.2	1.7	5.7	4.2	$Cd_{5639}Se_{216}S_{5423}$
	**h**	3.0	1.3	5.7	4.2	$Cd_{5639}Se_{504}S_{5135}$
	**i**	3.9	0.9	5.7	4.2	$Cd_{5639}Se_{966}S_{4673}$

Supplementary Table 1: Dimensions of QD dimers (in nm) by controlling D_{core} and D_{neck}. The total dimension $D = D_{\text{core}} + 2D_{\text{shell}}$.

Orientations	Label	D_{core}	D_{shell}	D	D_{neck}	Formula
[100]	**a**	2.2	1.4	5.0	2.5	$Cd_{3812}Se_{216}S_{3569}$
	**b**	3.0	1.3	5.7	2.9	$Cd_{5330}Se_{504}S_{4826}$
	**c**	3.9	1.2	6.3	3.3	$Cd_{7226}Se_{966}S_{6260}$
	**d**	2.2	1.1	4.3	2.1	$Cd_{2630}Se_{216}S_{2414}$
	**e**	3.0	0.9	4.7	2.5	$Cd_{3660}Se_{504}S_{3156}$
	**f**	3.9	0.9	5.7	2.9	$Cd_{5330}Se_{966}S_{4364}$
	**g**	2.2	0.7	3.6	1.4	$Cd_{1742}Se_{216}S_{1523}$
	**h**	3.0	0.7	4.3	2.1	$Cd_{2478}Se_{504}S_{1974}$
	**i**	3.9	0.6	5.0	2.5	$Cd_{3812}Se_{966}S_{2846}$
[001]	**a**	2.2	1.4	5.0	2.5	$Cd_{3660}Se_{216}S_{3444}$
	**b**	3.0	1.3	5.7	2.9	$Cd_{5202}Se_{504}S_{4698}$
	**c**	3.9	1.2	6.3	3.3	$Cd_{7125}Se_{966}S_{6159}$
	**d**	2.2	1.1	4.3	2.1	$Cd_{2457}Se_{216}S_{2241}$
	**e**	3.0	0.9	4.7	2.5	$Cd_{3660}Se_{504}S_{3156}$
	**f**	3.9	0.9	5.7	2.9	$Cd_{5202}Se_{966}S_{4236}$
	**g**	2.2	0.7	3.6	1.4	$Cd_{1545}Se_{216}S_{1329}$
	**h**	3.0	0.7	4.3	2.1	$Cd_{2457}Se_{504}S_{1953}$
	**i**	3.9	0.6	5.0	2.5	$Cd_{3660}Se_{966}S_{2694}$

Supplementary Table 2: Dimensions of QD dimers (in nm) by controlling D_{core} and D_{shell}. The total dimension $D = D_{\text{core}} + 2D_{\text{shell}}$.

Supplementary note 2: Quasi-electron states calculation and localization

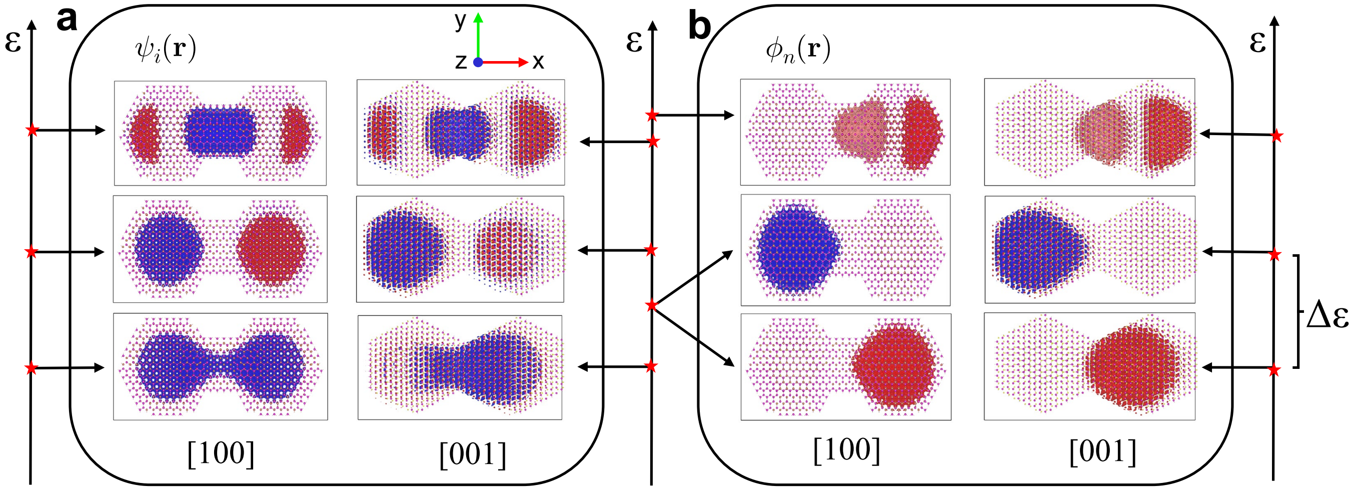

Supplementary Figure 2: Hyper-sphere plots of quasi-electron eigenstates and local states. **a**, **b** Quasi-electron eigenstates $\psi_i(\mathbf{r})$ (**a**) and local states $\phi_n(\mathbf{r})$ (**b**) are obtained from the semi-empirical pseudopotential calculation and FB-localization for QD dimers in symmetric [100] and asymmetric [001] attachment orientations. Blue and red denote positive and negative phases of the wavefunction in the eigenstates and denote donor and acceptor states in the local states. For the asymmetric orientation, there exists an energy bias $\Delta\varepsilon$ between the donor and acceptor states.

The semi-empirical pseudopotential model [3, 4] was used to describe the quasi-electron Hamiltonian and the filter diagonalization technique [5, 6] was applied to calculate the eigenstates of the dimer near the bottom of the conduction band. The local screened strain-dependent pseudopotentials following Wang *et al.* [7] were chosen the functional form in the momentum space

$$\tilde{\nu}(q) = a_0 \left[1 + a_4 \operatorname{Tr} \boldsymbol{\epsilon}\right] \frac{q^2 - a_1}{a_2 \exp\left(a_3 q^2\right) - 1} \tag{S1}$$

where q is the momentum, $\boldsymbol{\epsilon}$ is the strain tensor, and the pseudopotential parameters for Cd, Se, and S were collected in Table S3. All parameters were fitted to reproduce bulk band structures, band gaps, and effective masses of CdSe and CdS. The real-space quasi-electron Hamiltonian $\hat{h}_{\mathrm{QP}}(\mathbf{r})$ was given by

$$\hat{h}_{\mathrm{QP}}(\mathbf{r}) = -\frac{1}{2}\nabla_{\mathbf{r}}^2 + \sum_{\mu} \nu_{\mu}\left(|\mathbf{r} - \mathbf{R}_{0,\mu}|\right), \tag{S2}$$

where ν_μ is the real-space pseudopotential for atom μ. The filter-diagonalization technique was then applied to obtain quasiparticle electron states $\psi_i(\mathbf{r})$ above the conduction band edge (first 3 states shown in Supplementary Figure 2). The calculations were implemented on real-space grids less than 0.8 a.u. such that the eigenenergies converge less than 10^{-3} meV.

To transform the delocalized quasi-electron eigenstates $\psi_i(\mathbf{r})$ to localized donor and acceptor states $\phi_n(\mathbf{r})$, the

	a_1	a_2	a_3	a_4
Cd	-39.5761	1.0205	-0.1361	1.6688
Se	5.7389	4.3792	1.2123	0.3197
S	5.4875	4.3685	1.2135	0.2915

Supplementary Table 3: Pseudopotential parameters in Eq. S1 for Cd, Se, and S atoms. All parameters are given in atomic units.

Förster-Boys localization scheme was applied to maximize the self-extension criteria [8, 9]

$$\left\langle \hat{\Omega} \right\rangle_{FB} = \sum_{n \in \mathcal{D},\mathcal{A}} \left(\int \mathrm{d}^3\mathbf{r} |\phi_n(\mathbf{r})|^2 \mathbf{r} \right)^2, \tag{S3}$$

where the optimization can be achieved by successive 2×2 rotations of wavefunction pairs. The resulting localized states are related to eigenstates by a unitary matrix U

$$\phi_n(\mathbf{r}) = \sum_i U_{ni} \psi_i(\mathbf{r}). \tag{S4}$$

The first 8 eigenstates at the bottom of the conduction band were included to construct localized states.

Supplementary note 3: Population dynamics for individual states

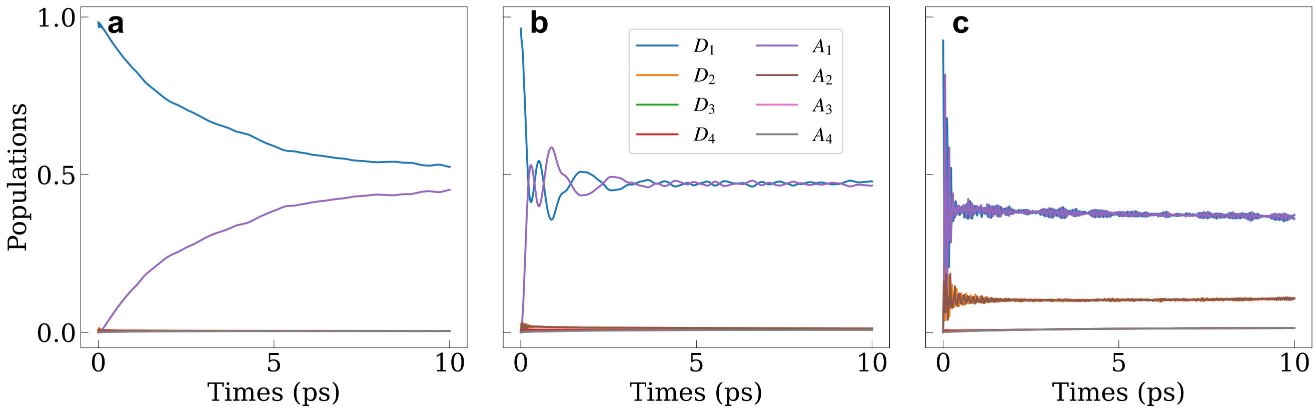

Supplementary Figure 3: Multi-state population dynamics. **a-c** Population dynamics for all 8 states (4 donor states labeled $D_n, n = 1 \ldots 4$ and 4 acceptor states labeled $A_m, m = 1 \ldots 4$) in the nonadiabatic (**a**), intermediate (**b**), and adiabatic (**c**) regimes as shown in Fig. 3 in the main text.

As discussed in the main text, 4 donor states and 4 acceptor states were taken to construct the Hamiltonian. The population dynamics for individual states corresponding to Fig. 3 in the main text are shown in Supplementary Figure 3. The population dynamics in Supplementary Figure 3a, b are dominated by the two $1S_e$-like ground states D_1 and A_1. This is because the energy gap between $1S_e$ and $1P_e$ is much greater than the thermal energy at 300 K in these two cases, so $1P_e$-like states are not populated according to the initial condition Eq. 15 in the main text.

On the other hand, the $1S_e$-$1P_e$ energy gap is smaller in the case of Supplementary Figure 3c, and therefore D_2 is partially populated. The population transfer between $1S_e$ and $1P_e$-like states will thus influence the total dynamics, including the dephasing rate of the ground states.

Supplementary note 4: Population dynamics for all structures

Supplementary Figures 4 to 7 plot the population dynamics of all the QD dimers with various D_{neck}, D_{core} and D_{shell} studied in the main text. The detailed dimension for each structure can be found in Table. S1 and S2 indexed by the corresponding label. Atomic coordinates for the relaxed structures can be found in Supplementary Data files. $\Delta\varepsilon$, J and λ are the energy bias, hybridization energy, and reorganization energy between two ground states (two $1S_e$-like D_1 and A_1 states). All calculations are performed at room temperature with temperature $T = 300$ K.

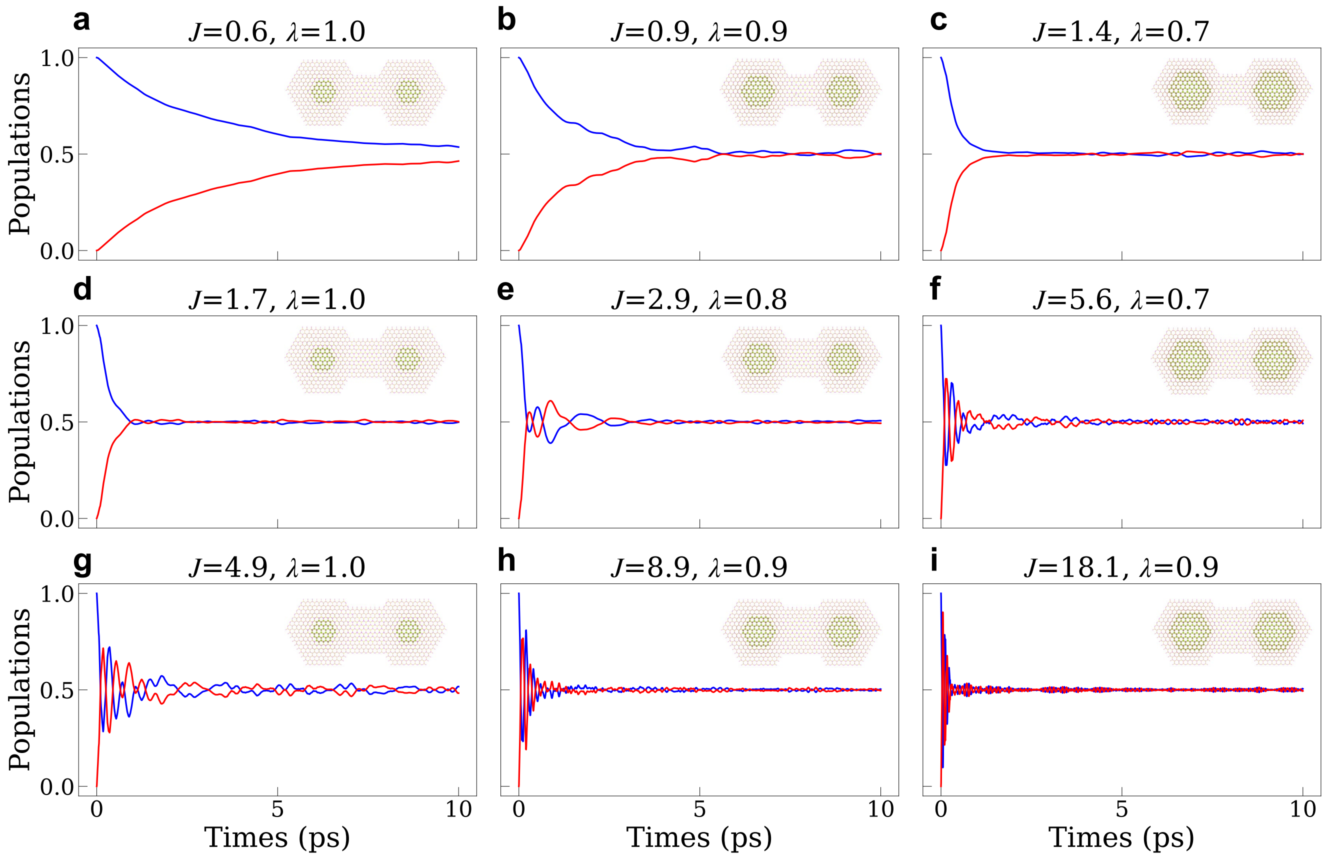

Supplementary Figure 4: Population dynamics of the QD dimers controlled by core diameters and neck width with symmetric [100] attachment. **a-i** Population dynamics of donor $p_{\mathcal{D}}(t)$ (blue) and acceptor $p_{\mathcal{A}}(t)$ (red) at 300 K for different core diameters D_{core} (columns: 2.2, 3.0, 3.9 nm) and neck width D_{neck} (rows: 2.4, 3.1, 3.8 nm). Check Table. S1 with corresponding labels for the detailed dimensions. J and λ label ground state hybridization energy and reorganization energy, respectively. The units for all parameters are in meV. The comparison between population dynamics shown in the main text Fig. 3 is taken from panels **a**, **e** and **i**. Source data are provided as a Source Data file.

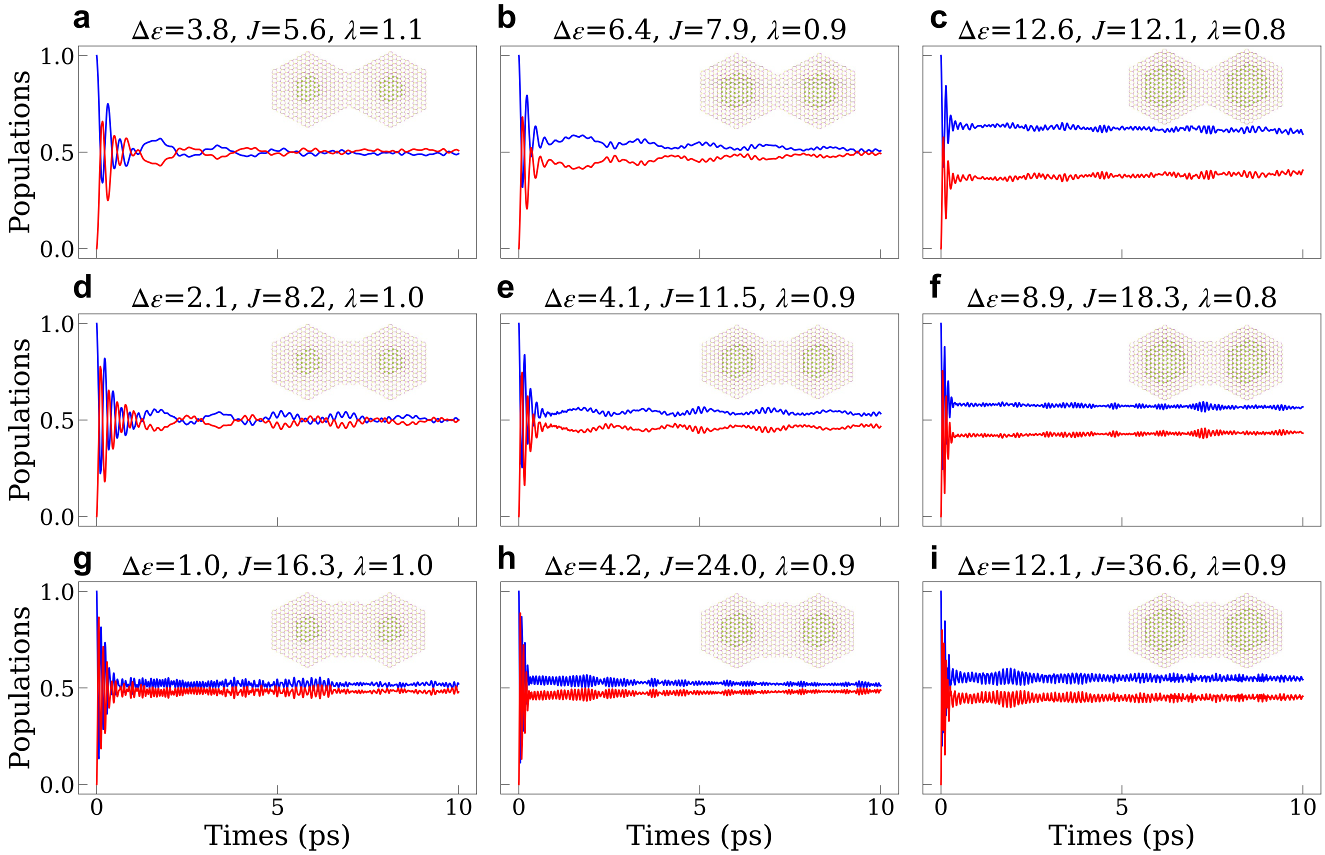

Supplementary Figure 5: Population dynamics of the QD dimers controlled by core diameters and neck width with asymmetric [001] attachment. a-i Population dynamics of donor $p_{\mathcal{D}}(t)$ (blue) and acceptor $p_{\mathcal{A}}(t)$ (red) at 300 K for different core diameters $D_{\rm core}$ (columns: 2.2, 3.0, 3.9 nm) and neck width $D_{\rm neck}$ (rows: 2.8, 3.5, 4.2 nm). Check Table. S1 with corresponding labels for the detailed dimensions. $\Delta\varepsilon$, J and λ label ground state driving force, hybridization energy and reorganization energy, respectively. The units for all parameters are in meV. Source data are provided as a Source Data file.

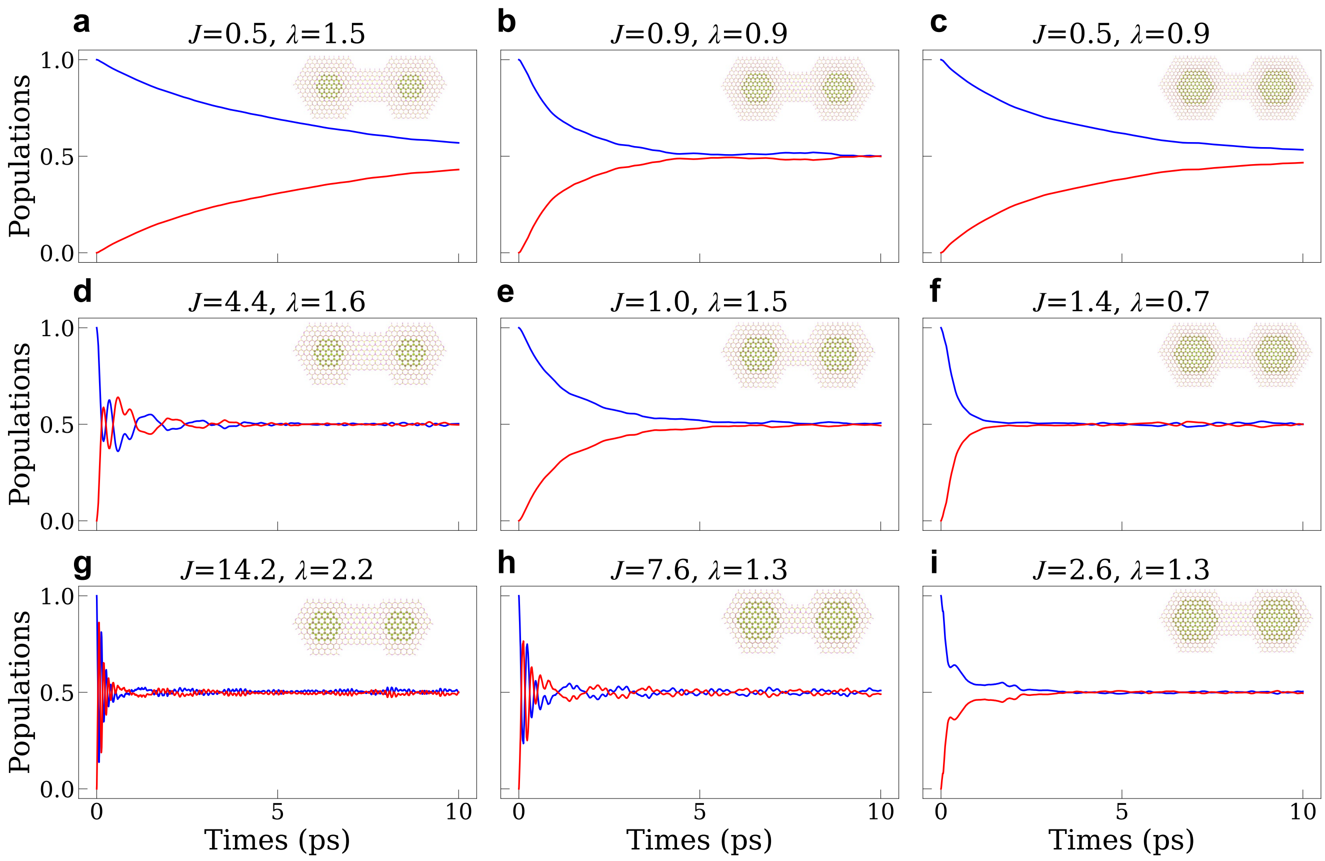

Supplementary Figure 6: Population dynamics of the QD dimers controlled by core diameters and shell thicknesses with symmetric [100] attachment. a-i Population dynamics of donor $p_{\mathcal{D}}(t)$ (blue) and acceptor $p_{\mathcal{A}}(t)$ (red) at 300 K for different core diameters D_{core} (columns: 2.2, 3.0, 3.9nm) and shell thicknesses D_{shell} (rows: 5-layer, 4-layer, 3-layer). Check Table. S2 with corresponding labels for the detailed dimensions. J and λ label ground state hybridization energy and reorganization energy, respectively. The units for all parameters are in meV. Source data are provided as a Source Data file.

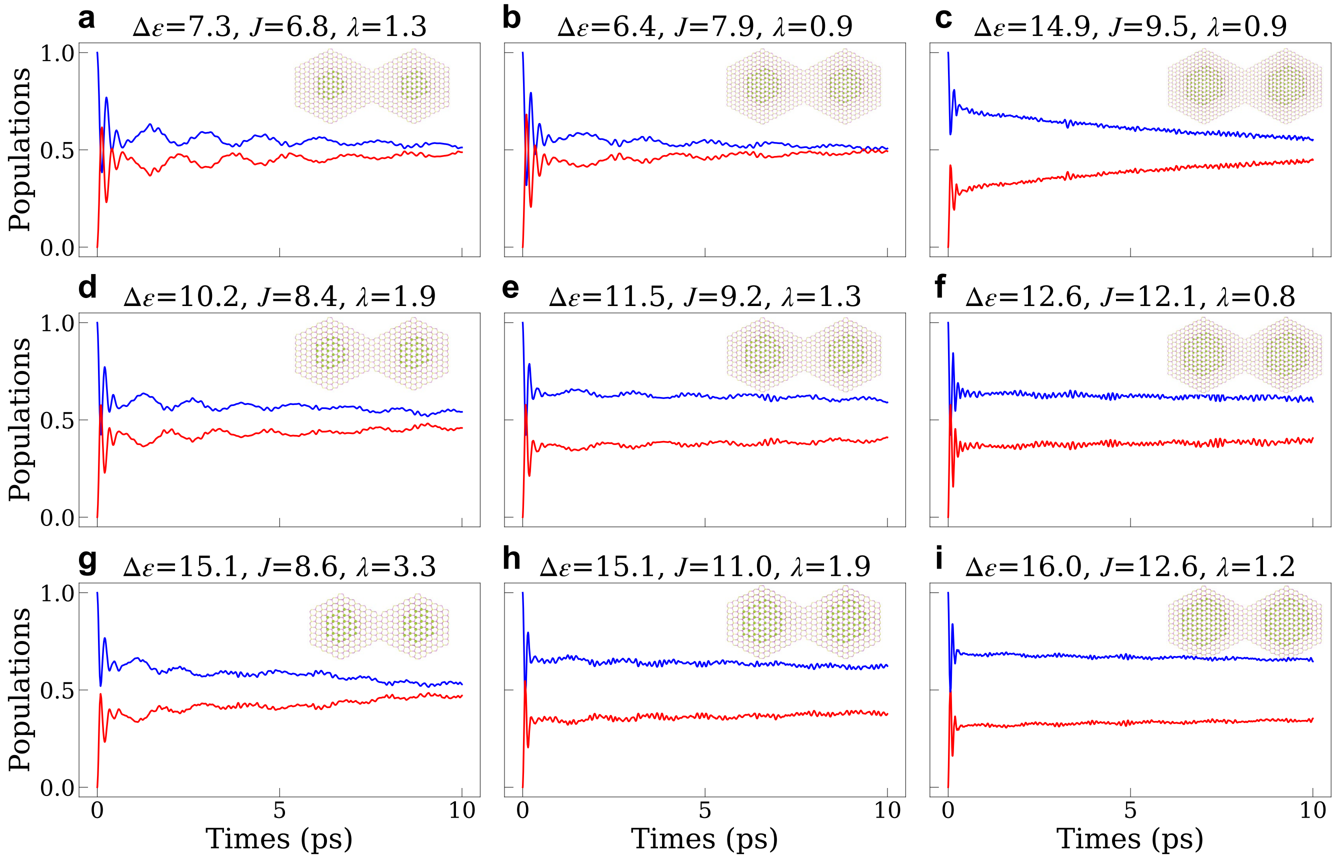

Supplementary Figure 7: Population dynamics of the QD dimers controlled by core diameters and shell thicknesses with asymmetric [001] attachment. a-i Population dynamics of donor $p_{\mathcal{D}}(t)$ (blue) and acceptor $p_{\mathcal{A}}(t)$ (red) at 300 K for different core diameters D_{core} (columns: 2.2, 3.0, 3.9nm) and shell thicknesses D_{shell} (rows: 5-layer, 4-layer, 3-layer). Check Table. S2 with corresponding labels for the detailed dimensions. $\Delta\varepsilon$, J and λ label ground state driving force, hybridization energy and reorganization energy, respectively. The units for all parameters are in meV. Source data are provided as a Source Data file.

Supplementary note 5: Validation of mean-field dynamics

The performance of mean-field Ehrenfest dynamics can be validated by numerically exact methods such as the multi-configurational time-dependent Hartree (MCTDH) approach [10–13]. To validate our mixed-quantum classical Ehrenfest approach applied in electron transfer, we simplify the original multi-level electronic system to a two-level system including only the ground donor and acceptor states. The vibrational modes are sampled from the spectral density (see Eq. 5 in the main text) based on the coupling strength. The reason we consider a simpler problem here is that MCTDH is very computationally expensive and sometimes infeasible for multi-level systems coupled with a large number of vibrational modes. However, this is not such a big simplification for systems where the ground donor and acceptor state dynamics dominate. The MCTDH calculations performed here for validation were implemented with the Heidelberg MCTDH package [14]. Supplementary Figure 8 shows the comparison of population dynamics calculated from Ehrenfest and MCTDH approach in nonadiabatic ($\gamma \ll 1$), intermediate ($\gamma \approx 1$), and adiabatic ($\gamma \gg 1$) regimes with only ground donor and acceptor states. The two methods agree very well across all electron transfer regimes in the parameter space we considered. In the nonadiabatic regime, Ehrenfest and MCTDH also agree with the exponential decay with transition rates obtained from Marcus theory shown in Supplementary Figure 8a. Supplementary Figure 8c validates the adiabatic dynamics in asymmetrically attached QD dimers where an energy bias $\Delta\varepsilon$ is present.

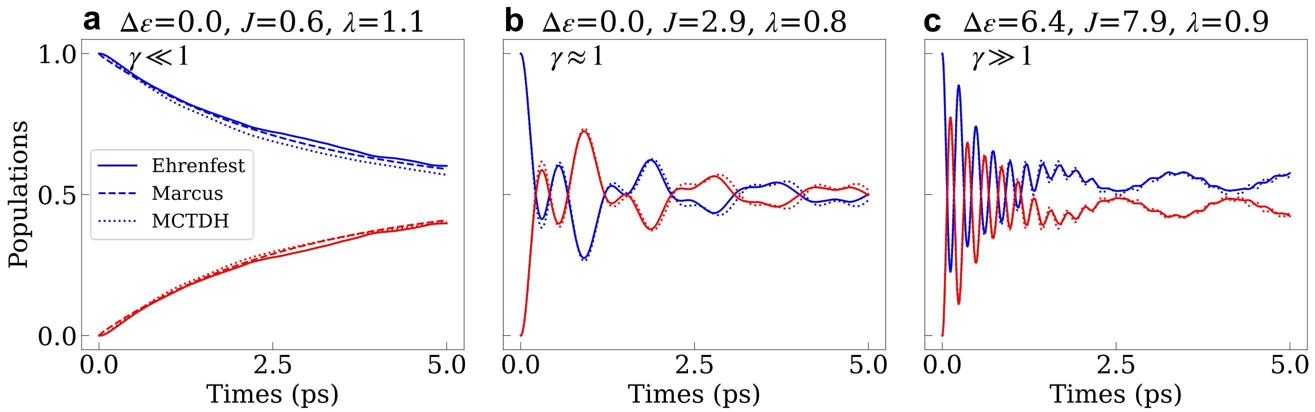

Supplementary Figure 8: Validation of the Ehrenfest population dynamics using MCTDH method across different regimes. **a-c** Comparison of population dynamics in the nonadiabatic (**a**), intermediate (**b**), and adiabatic (**c**) regimes, characterizing by the adiabatic parameter γ. Dynamics generated by Ehrenfest mean-field, Marcus master equation and MCTDH are shown in solid, dashed and dotted lines. Ground-state two-level dynamics are extracted from the multi-level dynamics shown in Supplementary Figure 4a, Supplementary Figure 4e and Supplementary Figure 5b, respectively. $\Delta\varepsilon$, J and λ label ground state driving force, hybridization energy and reorganization energy. The units for all parameters are in meV.

Supplementary note 6: Physical source of dephasing

Coupling to low-frequency vibrations. As shown in Supplementary Figure 4a of the main text, the spectral density $S_{D_1 D_1}(\omega)$ peaks at around 0.6 THz, which means that the electronic system couples most strongly to the low-frequency vibrations at room temperature. Those low-frequency vibrations contribute to the time scale of dephasing in the population dynamics. Supplementary Figure 9 compares the population dynamics with and without the vibrational modes below the characteristic frequency $\omega_c/2\pi = 0.6$ THz, which shows that the dephasing time becomes much longer when removing those low-frequency modes.

Effects of off-diagonal couplings. In the main text, we argued that the off-diagonal couplings along with population transfer between $1S_e$-like (D_1, A_1) and $1P_e$-like (D_2, A_2) states are responsible for the dephasing trend seen in the adiabatic regime. Supplementary Figure 10 shows the comparison between three adiabatic dynamics when including both diagonal and off-diagonal vibronic couplings (a-c) and when including only diagonal couplings (d-f). Note that the dephasing rate k_{dp} decreases significantly when the off-diagonal couplings are removed, yielding longer population coherence.

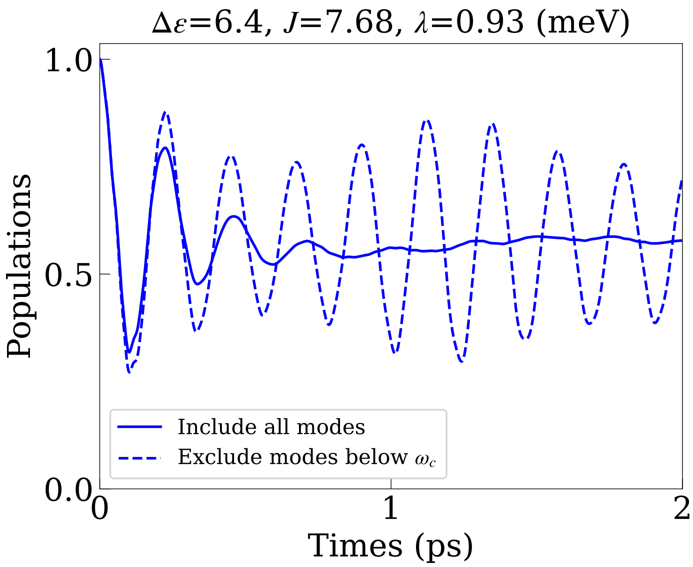

Supplementary Figure 9: Population dynamics without low-frequency modes. Comparison of the dynamics with (solid line) and without (dashed line) low-frequency modes below ω_c. The original dynamics is from Supplementary Figure 5b. $\Delta\varepsilon$, J and λ label ground state driving force, hybridization energy and reorganization energy, respectively. The units for all parameters are in meV.

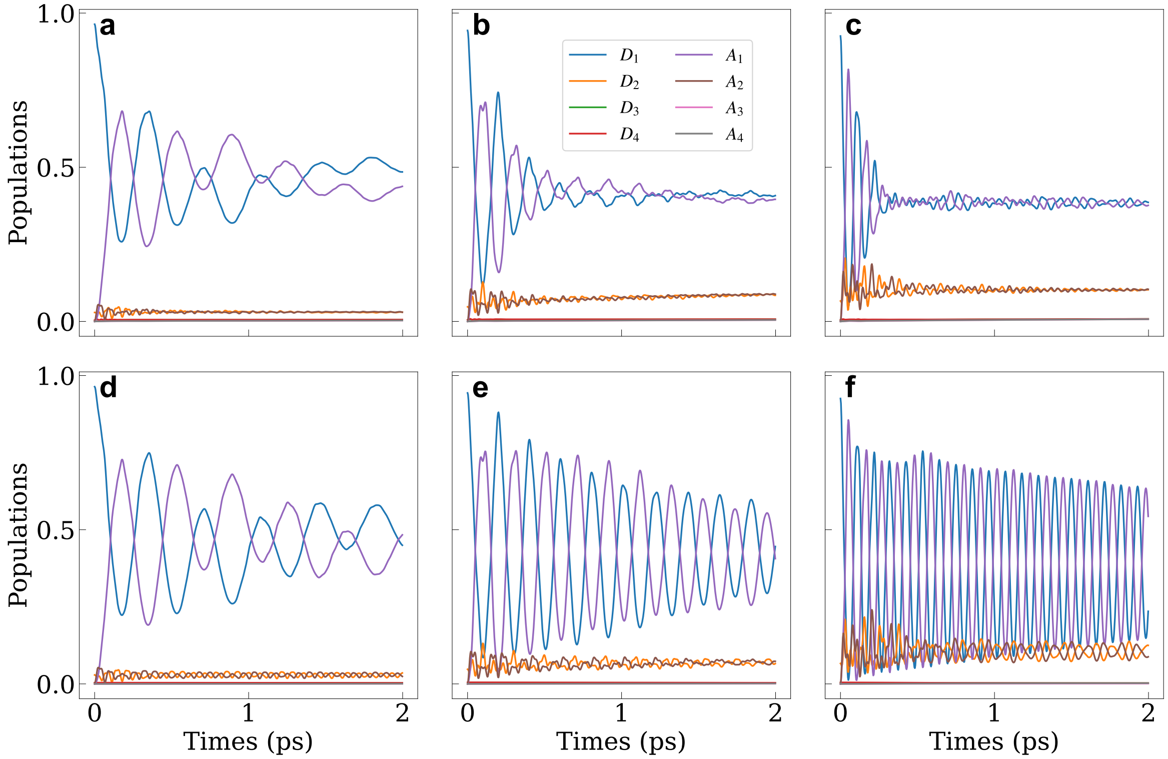

Supplementary Figure 10: Effects of off-diagonal couplings. a-c Population of individual states for the same QD dimer systems shown in Supplementary Figure 4g-h respectively. Both diagonal couplings V^{α}_{nn} and off-diagonal couplings $V^{\alpha}_{n\neq m}$ are included. **d-f** Population dynamics of the same systems as a-c but with off-diagonal couplings $V^{\alpha}_{n\neq m}$ removed.

Supplementary References

[1] Zhou, X. W. *et al.* Stillinger-Weber potential for the II-VI elements Zn-Cd-Hg-S-Se-Te. *Phys. Rev. B* **88**, 085309 (2013).

[2] Plimpton, S. Fast Parallel Algorithms for Short-Range Molecular Dynamics. *J. Comput. Phys.* **117**, 1–19 (1995).

[3] Wang, L. W. & Zunger, A. Electronic Structure Pseudopotential Calculations of Large (.apprx.1000 Atoms) Si Quantum Dots. *J. Phys. Chem.* **98**, 2158–2165 (1994).

[4] Rabani, E., Hetényi, B., Berne, B. J. & Brus, L. E. Electronic properties of CdSe nanocrystals in the absence and presence of a dielectric medium. *J. Chem. Phys.* **110**, 5355–5369 (1999).

[5] Wall, M. R. & Neuhauser, D. Extraction, through filter-diagonalization, of general quantum eigenvalues or classical normal mode frequencies from a small number of residues or a short-time segment of a signal. I. Theory and application to a quantum-dynamics model. *J. Chem. Phys.* **102**, 8011–8022 (1995).

[6] Toledo, S. & Rabani, E. Very Large Electronic Structure Calculations Using an Out-of-Core Filter-Diagonalization Method. *J. Comput. Phys.* **180**, 256–269 (2002).

[7] Wang, L.-W., Kim, J. & Zunger, A. Electronic structures of [110]-faceted self-assembled pyramidal InAs/GaAs quantum dots. *Phys. Rev. B* **59**, 5678–5687 (1999).

[8] Foster, J. M. & Boys, S. F. Canonical Configurational Interaction Procedure. *Rev. Mod. Phys.* **32**, 300–302 (1960).

[9] Kleier, D. A., Halgren, T. A., Hall, J. H. & Lipscomb, W. N. Localized molecular orbitals for polyatomic molecules. I. A comparison of the Edmiston-Ruedenberg and Boys localization methods. *J. Chem. Phys.* **61**, 3905–3919 (1974).

[10] Meyer, H. D., Manthe, U. & Cederbaum, L. S. The multi-configurational time-dependent Hartree approach. *Chemical Physics Letters* **165**, 73–78 (1990).

[11] Manthe, U., Meyer, H. & Cederbaum, L. S. Wave-packet dynamics within the multiconfiguration Hartree framework: General aspects and application to NOCl. *The Journal of Chemical Physics* **97**, 3199–3213 (1992).

[12] Beck, M. H., Jäckle, A., Worth, G. A. & Meyer, H. D. The multiconfiguration time-dependent Hartree (MCTDH) method: a highly efficient algorithm for propagating wavepackets. *Physics Reports* **324**, 1–105 (2000).

[13] Wang, H. & Thoss, M. Multilayer formulation of the multiconfiguration time-dependent Hartree theory. *J. Chem. Phys.* **119**, 1289–1299 (2003).

[14] Beck, M. H., Jäckle, A., Worth, G. A. & Meyer, H. D. The mctdh package, version 8.5 (2020). URL `http://mctdh.uni-hd.de/`.
